# Excessive Adventitial and Perivascular Vascularisation Correlates with Vascular Inflammation and Intimal Hyperplasia

**DOI:** 10.3390/ijms232012156

**Published:** 2022-10-12

**Authors:** Leo Bogdanov, Daria Shishkova, Rinat Mukhamadiyarov, Elena Velikanova, Anna Tsepokina, Alexander Terekhov, Vladislav Koshelev, Anastasia Kanonykina, Amin Shabaev, Alexey Frolov, Nikita Zagorodnikov, Anton Kutikhin

**Affiliations:** Department of Experimental Medicine, Research Institute for Complex Issues of Cardiovascular Diseases, 6 Sosnovy Boulevard, 650002 Kemerovo, Russia

**Keywords:** vasa vasorum, tunica adventitia, perivascular adipose tissue, vascular inflammation, intimal hyperplasia, coronary artery bypass graft surgery, internal mammary arteries, saphenous veins

## Abstract

Albeit multiple studies demonstrated that *vasa vasorum* (VV) have a crucial importance in vascular pathology, the informative markers and metrics of vascular inflammation defining the development of intimal hyperplasia (IH) have been vaguely studied. Here, we employed two rat models (balloon injury of the abdominal aorta and the same intervention optionally complemented with intravenous injections of calciprotein particles) and a clinical scenario (arterial and venous conduits for coronary artery bypass graft (CABG) surgery) to investigate the pathophysiological interconnections among VV, myeloperoxidase-positive (MPO^+^) clusters, and IH. We found that the amounts of VV and MPO^+^ clusters were strongly correlated; further, MPO^+^ clusters density was significantly associated with balloon-induced IH and increased at calciprotein particle-provoked endothelial dysfunction. Likewise, number and density of VV correlated with IH in bypass grafts for CABG surgery at the pre-intervention stage and were higher in venous conduits which more frequently suffered from IH as compared with arterial grafts. Collectively, our results underline the pathophysiological importance of excessive VV upon the vascular injury or at the exposure to cardiovascular risk factors, highlight MPO^+^ clusters as an informative marker of adventitial and perivascular inflammation, and propose another mechanistic explanation of a higher long-term patency of arterial grafts upon the CABG surgery.

## 1. Introduction

*Vasa vasorum* (VV) represent a microvascular network supplying the blood vessel wall with the oxygen and nutrients and concurrently providing a route for the immune cells to patrol adventitia and perivascular adipose tissue [1,2,3,4]. Through the vascular development, VV emerge in the blood vessels at distances exceeding the limit of oxygen and nutrients diffusion (0.5 mm, equal to 29 lamellar units in the elastic arteries) [1,2,3]. By origin, VV may have either arterial or venous specification [1,2,5]. Arterial VV branch from adjacent arteries (*vasa vasorum externa*) or stem from the vessel lumen and tunica media (i.e., *vasa vasorum interna*); however, most arterial VV are defined as *vasa vasorum externa* [1,2,5]. With respect to the blood vessel types, veins contain only *vasa vasorum externa* while arteries have both *vasa vasorum externa* and *vasa vasorum interna* [5], although the former significantly prevail [2]. Expectedly, venous VV are observed in both veins and arteries [1,2,3,5]. Several orders of VV have been observed, where first- and third-order VV are generally located longitudinally along the blood vessel in contrast with the second- and fourth-order VV which are split from them across the vessel wall [1,2,3,5]. The amount of VV varies considerably across the blood vessels and is generally higher in veins than in arteries [6]. Regarding the cellular composition, VV consist of endothelial cells and regularly layered vascular smooth muscle cells or pericytes, whereas first-order VV may also have a connective tissue layer reminiscent of the large vessels [2].

Albeit VV are indispensable for vascular physiology, a mutual interconnection between angiogenesis and inflammation propelled the seminal research on their role in vascular disease. Studies on hypercholesterolemic animals found that growth of VV networks precedes the development of the atherosclerotic lesions [7,8], and VV expansion is intimately associated with neointimal remodeling [9,10], while anti-angiogenic therapies inhibited intimal hyperplasia (IH) [11] and plaque growth [12,13,14]. In addition to VV, murine and rat adventitia and perivascular adipose tissue include myeloperoxidase-positive (MPO^+^) clusters indicative of vascular inflammation [15,16,17,18,19], yet an association between the development of MPO^+^ clusters and VV sprouting remains elusive. Further, in keeping with the correlation of adventitial and perivascular microvessels with vascular inflammation, leaky plaque neovessels (*vasa plaquorum*, VP) are well recognised as an important driver of atherosclerotic progression through intraplaque haemorrhages, increased lipid deposition, and enhanced macrophage migration [20,21,22,23,24,25,26,27]. Compared with VV, VP have a thin wall, incomplete endothelial junctions, detached basement membrane, and suffer from a tensile and thermal stress [28,29,30]. Similar to VP, the magnitude of adventitial and perivascular vascularisation is also associated with plaque volume, instability, and clinical manifestations [31,32,33,34,35,36,37], suggesting a pathogenetic link between plaque neovessels and pre-existing arterial vasculature. Intriguingly, VV and VP together provide a route converging inside-out and outside-in mechanisms of atherosclerosis, as both of them imply systemic endothelial dysfunction triggered by cardiovascular risk factors such as dyslipidaemia, arterial hypertension, overweight/obesity, diabetes mellitus, and chronic kidney disease [1,4,6]. Impairment of endothelial homeostasis augments vascular permeability, thereby promoting retention of ApoB-containing atherogenic lipoproteins, inducing extravasation of leukocytes into the neointima, and contributing to intraplaque haemorrhage [38,39,40,41]. Collectively, these results indicate the crucial importance of neointimal, adventitial, and perivascular microcirculation in vascular pathology.

Despite an established role of VV and VP in the development of vascular inflammation and atherosclerosis, the quantitative associations among VV, MPO^+^ clusters, and IH have been unclear to date, particularly in the context of endothelial dysfunction. Here, we revealed that the amounts of VV and MPO^+^ clusters are strongly correlated. Notably, MPO^+^ cluster density was particularly associated with balloon-induced IH of rat aortas, increased amounts of the immune cells in the tunica adventitia, and was augmented at endothelial dysfunction in this experimental model. Compared with VV, MPO^+^ clusters were more consistently associated with IH when assessed by several quantitative measures. In a clinical setting (patients who underwent coronary artery bypass graft (CABG) surgery), absolute number and density of VV correlated with pre-CABG IH in arterial and venous conduits and were higher in saphenous vein (SV) than in internal mammary artery (IMA) grafts. Together, these findings underscore the pathophysiological role of excessive adventitial and perivascular vascularisation and suggest additional mechanism behind the higher long-term patency of IMAs in comparison with SVs upon the CABG surgery.

## 2. Results

**Figure 1:**
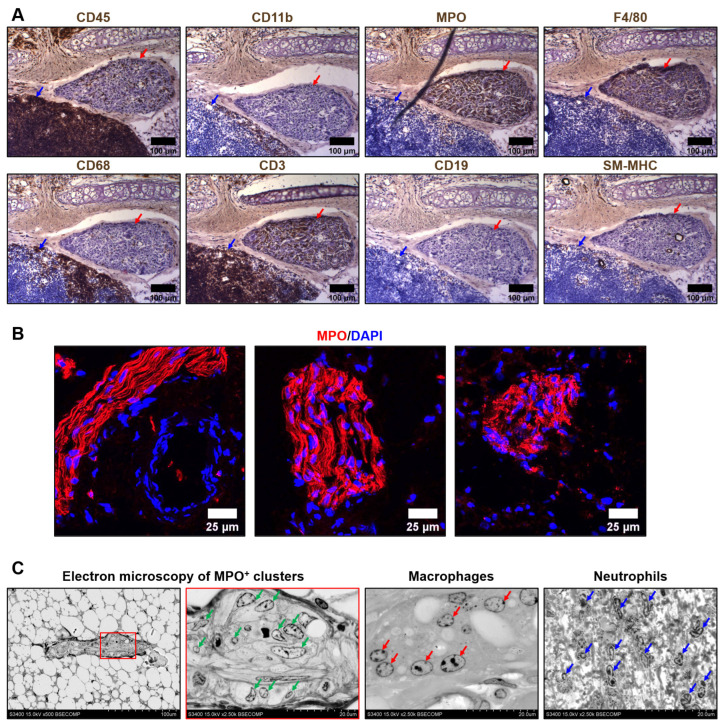
Immuno- and electron microscopy phenotyping of MPO^+^ clusters reveals positive staining for myeloperoxidase and macrophage appearance of the cells. (**A**) Immunohistochemical staining for a pan-leukocyte marker CD45, myeloid cell markers CD11b and myeloperoxidase (MPO), macrophage markers F4/80 and CD68, T cell marker CD3, B cell marker CD19, and vascular smooth muscle cell marker SM-MHC. Note the positive staining of MPO^+^ clusters (indicated by red arrows) for MPO, F4/80, and CD3. In contrast, the adjacent lymph node (indicated by blue arrows) is totally positive for CD45 and CD3. ×200 magnification (scale bar: 100 µm); (**B**) Verification immunofluorescence staining for MPO. Note the positive MPO^+^ clusters and a negative adjacent blood vessel (left image). Nuclei are counterstained with 4′,6-diamidino-2-phenylindole (DAPI). ×400 magnification (scale bar: 25 µm); and (**C**) EM-BSEM visualisation of MPO^+^ clusters. Note the similarity of the cells within the MPO^+^ clusters (indicated by green arrows) to macrophages (indicated by red arrows) but not neutrophils (indicated by blue arrows). The right MPO^+^ clusters image (×2500 magnification) is a close-up of the left image (×500 magnification). ×500–×2500 magnification (scale bar: 100 and 20 µm, accelerating voltage: 15 kV).

We first performed a comprehensive immunophenotyping of the cells composing the MPO^+^ clusters located in the tunica adventitia and perivascular adipose tissue of rat aortas. In contrast with the circulating leukocytes, resident cells within the MPO^+^ clusters were sparsely positive for a pan-leukocyte marker CD45 and were generally negative for a myeloid cell marker CD11b, instead being positive for another myeloid cell marker myeloperoxidase (MPO), a macrophage marker F4/80, and a T cell marker CD3 (Figure 1A). As MPO staining was the most consistent and prominent among distinct MPO^+^ clusters as compared with F4/80 and CD3 staining, we further tested anti-MPO antibodies as an instrument to demarcate MPO^+^ clusters in an immunofluorescence setting and successfully replicated the initial results (Figure 1B). In contrast, perivascular lymph nodes were MPO-negative (Figure S1). Next, we visualised MPO^+^ clusters by means of backscattered scanning electron microscopy upon epoxy resin embedding (EM-BSEM) of rat aorta. Albeit MPO is an established marker of both neutrophils and macrophages, cells within the MPO^+^ clusters resembled macrophages but not neutrophils (Figure 1C).

**Figure 2:**
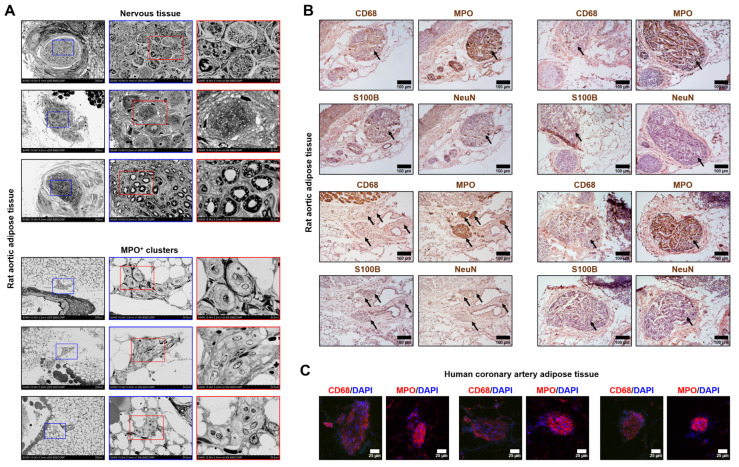
Ultrastructural examination of MPO^+^ clusters and nerve bundles. (**A**) Backscattered scanning electron microscopy examination of the different types of nerve bundles (top) and MPO^+^ clusters (bottom). Rat aortic adipose tissue. Red and blue contours demarcate ×1000 and ×2500 close-ups, respectively. ×250, ×1000, and ×2500 magnification (scale bars: 200 µm, 50 µm, and 20 µm, respectively; accelerating voltage: 15 kV); (**B**) Immunohistochemical staining of MPO^+^ clusters (indicated by black arrows) for a macrophage marker CD68, myeloid cell marker myeloperoxidase, and neural tissue markers S100B and NeuN. Rat aortic adipose tissue. Note that MPO^+^ clusters are positively stained for myeloperoxidase and CD68 while being negatively stained for S100B and NeuN. ×200 magnification (scale bar: 100 µm); and (**C**) Immunofluorescence anti-CD68 and anti-MPO staining of the human coronary artery adipose tissue. Note the positive red staining within the MPO^+^ clusters and negative staining around them. DAPI nuclear counterstaining. ×400 magnification (scale bar: 25 µm). DAPI nuclear counterstaining. MPO—myeloperoxidase, DAPI—4′,6-diamidino-2-phenylindole.

To distinguish MPO^+^ clusters from the nerve bundles, we conducted an ultrastructural examination and found that these structures have vastly different appearance (Figure 2A). To corroborate these observations, we stained MPO^+^ clusters for the neural tissue markers S100B and NeuN, revealing they are not expressed in MPO^+^ clusters, in contrast with MPO and a macrophage marker CD68 (Figure 2B). Next, we showed that human coronary artery adipose tissue also contains MPO- and CD68-positive clusters, indicating that these structures are not specific for rats and are also present in human tissue (Figure 2C). Intriguingly, expression of MPO notably prevailed over CD68 expression (Figure 2B,C). Albeit we still cannot exclude the possibility that MPO^+^ clusters consist of resident macrophages of the nervous system, we focus on their immune/pro-inflammatory phenotype (as shown by positive MPO/CD68 and negative S100B/NeuN staining) and not on other functions.

Then, we investigated whether IH (i.e., formation of neointima leading to vascular stenosis) is associated with excessive vascularisation and adventitial/perivascular inflammation which are, respectively, reflected by VV and MPO^+^ clusters. Having performed a balloon angioplasty of rat abdominal aorta, we carried out a histological investigation 5 weeks post-operation employing haematoxylin and eosin staining and found that IH has been developed in 20/40 (50%) animals. Number, area, and density were used as measures to quantify the amount of VV and MPO^+^ clusters. VV and MPO^+^ clusters density was calculated both by number (i.e., the area of adventitial and perivascular tissue per 1 VV or MPO^+^ cluster) and area (i.e., the area of adventitial and perivascular tissue per 1 µm^2^ VV or MPO^+^ clusters). Hence, the smaller the area of adventitial and perivascular tissue per 1 VV/ MPO^+^ cluster or 1 µm^2^ VV/ MPO^+^ clusters, the higher the VV/ MPO^+^ clusters density.

**Figure 3:**
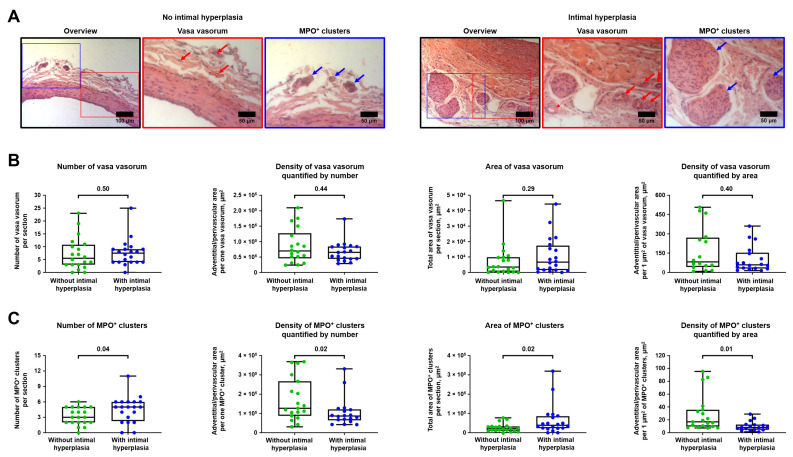
Balloon-induced IH is associated with the expansion of VV and development of MPO^+^ clusters. (**A**) Representative images showing VV and MPO^+^ clusters in rats with or without IH in the abdominal aortas 5 weeks upon the balloon angioplasty. Red and blue contours demarcate the close-ups of the areas with representative VV and MPO^+^ clusters, respectively. Red and blue arrows indicate VV and MPO^+^ clusters, respectively. Haematoxylin and eosin staining, ×200 magnification (scale bar: 100 µm) and ×400 magnification (scale bar: 50 µm); (**B**) Quantitative analysis of number, area, and density of VV in rats from the experiment in A. Each dot represents an aortic section from one rat. Whiskers indicate range, box bounds indicate 25th–75th percentiles, center lines indicate median. *p*-values provided above boxes, Mann–Whitney U-test; and (**C**) Quantitative analysis of number, area, and density of MPO^+^ clusters in rats from the experiment in A. Each dot represents an aortic section from one rat. Whiskers indicate range, box bounds indicate 25th–75th percentiles, center lines indicate median. *p*-values provided above boxes, Mann–Whitney U-test. IH—intimal hyperplasia, VV—vasa vasorum, and MPO—myeloperoxidase.

Count and area were highly concordant in both VV and MPO^+^ clusters (Spearman’s r = 0.58 and 0.72, respectively) that approved the use of these metrics for the quantitative analysis (Figure S2). Numbers of VV and MPO^+^ clusters were also well correlated (Spearman’s r = 0.35, Figure S2), in contrast with their areas (Spearman’s r = 0.17, Figure S2). Increased density of VV failed to demonstrate a statistically significant association with IH, whilst all metrics of MPO^+^ clusters were significantly associated with IH (Figure 3A–C). In keeping with these findings, both area and density of MPO^+^ clusters, but not VV, correlated with the amount of IH expressed as neointima-to-intima ratio or percent stenosis (MPO^+^ clusters, Spearman’s r = 0.46–0.50 for area and from −0.52 to −0.53 for density, Figure S3A; VV, Spearman’s r = 0.21–0.24 for area and from −0.20 to −0.23 for density, Figure S3B).

**Figure 4:**
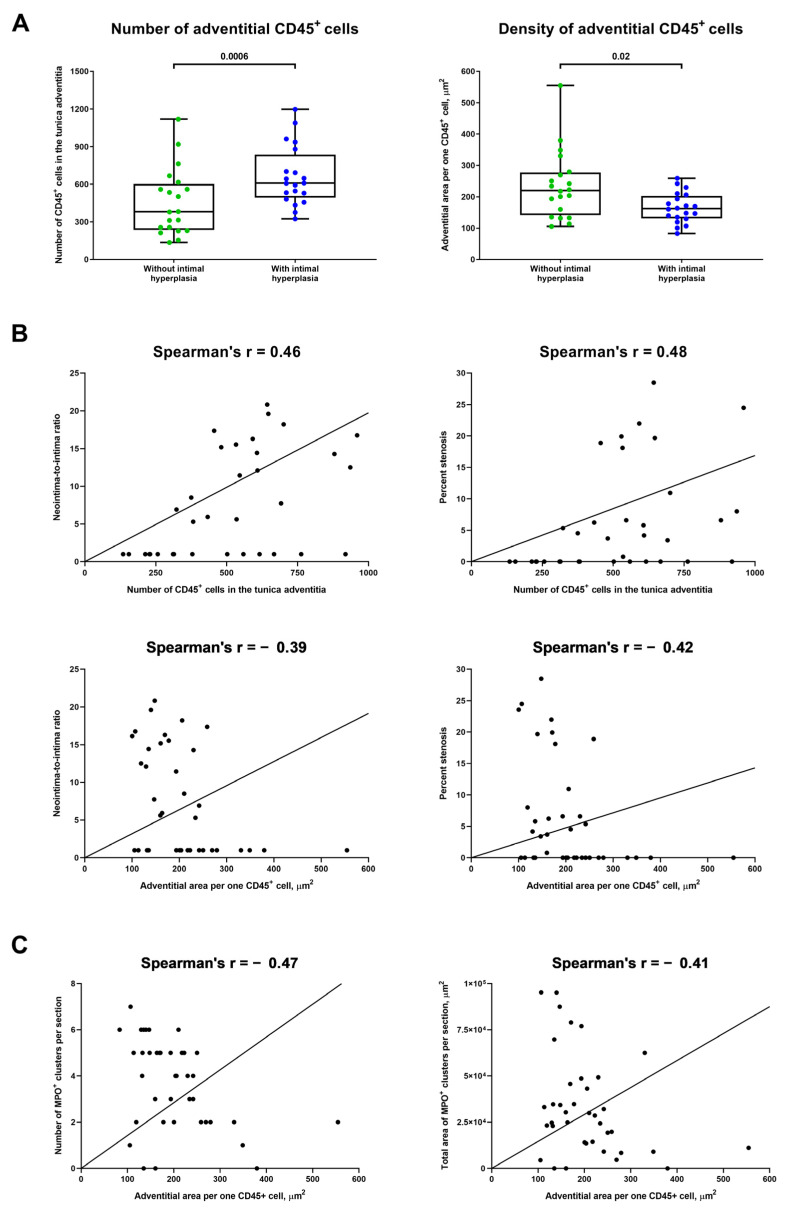
Number and density of immune cells are associated with the development of IH and expansion of MPO^+^ clusters. (**A**) Quantitative analysis of number and density of CD45^+^ cells in the tunica adventitia of rats with or without IH in the abdominal aortas 5 weeks upon the balloon angioplasty (from the experiment in Figure 3A). Each dot represents an aortic section from one rat. Whiskers indicate range, box bounds indicate 25th–75th percentiles, center lines indicate median. *p*-values provided above boxes, Mann–Whitney U-test; (**B**) Correlation plots indicate a strong correlation of the number of CD45^+^ cells (top) and their density (bottom) in the tunica adventitia with both measures of intimal hyperplasia (neointima-to-intima ratio, left and percent stenosis, right). Spearman’s rank correlation coefficient; and (**C**) Correlation plots showing a moderate and statistically significant correlation between the density of CD45^+^ cells in the tunica adventitia and both number (left) and area (right) of MPO^+^ clusters. Spearman’s rank correlation coefficient. MPO—myeloperoxidase, VV—vasa vasorum, and IH—intimal hyperplasia.

For deeper analysis of the interrelations between different mechanisms of adventitial inflammation upon vascular injury, we also analysed whether immune cell numbers and density in the tunica adventitia are associated with MPO^+^ expansion. Both number and density of CD45^+^ cells in the tunica adventitia were considerably higher in rats with IH (Figure 4A) and well correlated with neointima-to-intima ratio and percent stenosis (Spearman’s r = 0.46–0.48 for number and from −0.39 to −0.42 for density, Figure 4B). Further, density of CD45^+^ cells significantly correlated with both number and area of MPO^+^ clusters (Spearman’s r = −0.47 and −0.41, respectively, Figure 4C). Therefore, we concluded that number and density of immune cells are associated with the expansion of MPO^+^ clusters and the development of IH, underscoring the intricate balance between vascular inflammation and neointimal formation.

To investigate the mechanism of the above-mentioned associations, we complemented the balloon angioplasty of rat abdominal aorta with daily intravenous injections of CPPs, a newly discovered trigger of endothelial dysfunction [42,43,44], for 5 days. Control rats were subjected to balloon injury alone. Four weeks after the last injection, rats were euthanised and their aortas were examined using EM-BSEM to better distinguish between adventitial and perivascular VV. Similar to the previous experimental model, count and area were concordant in both VV and MPO^+^ clusters (Spearman’s r = 0.94 and 0.82, respectively), and both numbers and areas of VV and MPO^+^ clusters were also significantly correlated (Spearman’s r = 0.66 and 0.47, respectively, Figure S4A). Numbers and areas of adventitial VV and MPO^+^ clusters correlated more strongly (Spearman’s r = 0.55 for both metrics) than those in perivascular adipose tissue (Spearman’s r = 0.38 and 0.32, respectively, Figure S4B).

**Figure 5:**
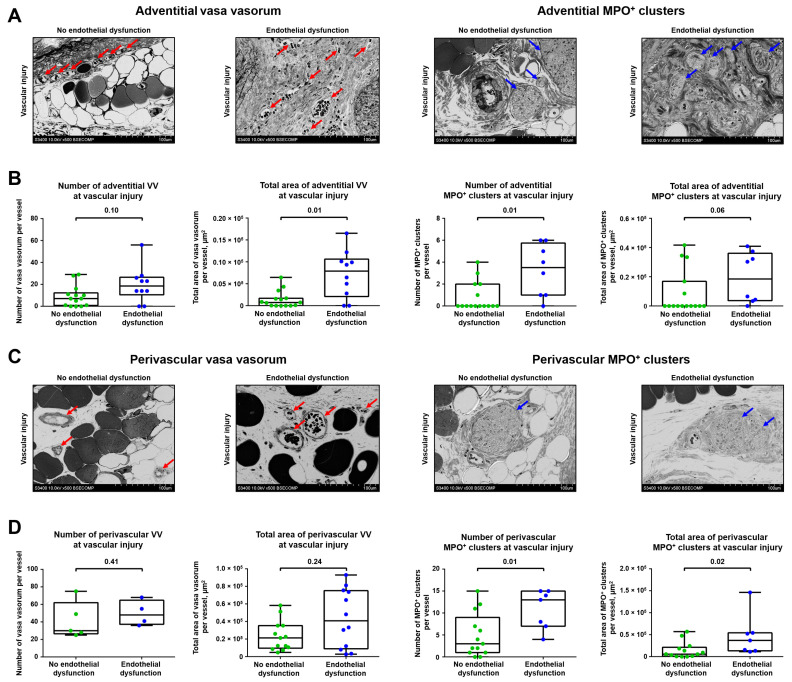
Endothelial dysfunction enhances sprouting of VV and growth of MPO^+^ clusters. (**A**) Representative images showing adventitial VV and MPO^+^ clusters in rats which underwent balloon angioplasty of the abdominal aorta and optional intravenous administration of CPPs, a trigger of endothelial dysfunction (900 µL of particles per injection, equal to 600 µg calcium) or equal volume of physiological saline for 5 days, followed by euthanasia upon 5 weeks. Red and blue arrows indicate VV and MPO^+^ clusters, respectively. EM-BSEM, ×500 magnification (scale bar: 100 µm, accelerating voltage: 10 kV); (**B**) Quantitative analysis of number and area of adventitial VV and MPO^+^ clusters in rats from the experiment in A. Each dot represents a cross-section of the epoxy resin-embedded aorta. Whiskers indicate range, box bounds indicate 25th–75th percentiles, center lines indicate median. *p*-values provided above boxes, Mann–Whitney U-test; (**C**) Representative images showing perivascular VV and MPO+ clusters in rats from the experiment in A. Red and blue arrows indicate VV and MPO^+^ clusters, respectively. EM-BSEM, ×500 magnification (scale bar: 100 µm, accelerating voltage: 10 kV); and (**D**) Quantitative analysis of number and area of perivascular VV and MPO^+^ clusters in rats from the experiment in (**A**). Each dot represents a cross-section of the epoxy resin-embedded aorta. Whiskers indicate range, box bounds indicate 25th–75th percentiles, center lines indicate median. *p*-values provided above boxes, Mann–Whitney U-test. VV—vasa vasorum, MPO—myeloperoxidase, CPPs—calciprotein particles, and EM-BSEM—embedding and backscattered scanning electron microscopy.

In comparison with the aortas of control rats, blood vessels of animals which received injections of CPPs had higher area of adventitial VV and more adventitial MPO^+^ clusters at the site of vascular injury, although number of adventitial VV and area of adventitial MPO^+^ clusters also tended to show statistically significant associations (Figure 5A,B). However, only MPO^+^ cluster-related metrics were increased in perivascular adipose tissue of the study group (Figure 5C,D). Collectively, enlarged VV and increased MPO^+^ clusters in aortas exposed to CPPs in addition to the existing balloon injury suggested a putative role of endothelial dysfunction in the development of VV networks and adventitial/perivascular inflammation.

Finally, we evaluated whether the excessive adventitial/perivascular vascularisation is associated with IH in a clinical scenario. To test the indicated hypothesis, we selected CABG surgery conduits, as this intervention is typically performed in patients > 50 years of age having several comorbid conditions (Table 1); moreover, the most frequent grafts are IMAs and SVs which undergo age-related remodeling [6,45,46]. Thus, we pairwise collected segments of 30 IMAs and SVs used as the first and the second grafts, respectively, for CABG surgery, and then investigated the amount of pre-implantation IH as well as number, area, and density of VV.

**Figure 6:**
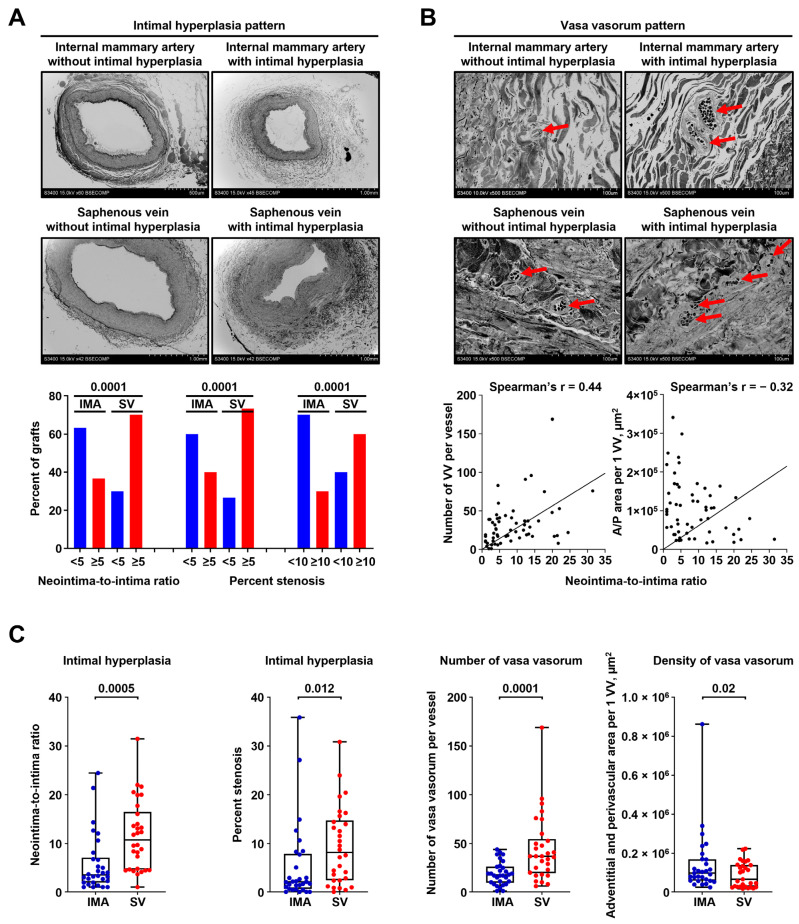
Saphenous veins utilised for CABG surgery frequently suffer from pre-implantation stenosis which correlates with the amount of VV. (**A**) Above: representative images showing pairwise collected conduits for CABG surgery (IMAs, top and SVs, bottom) without (left) or with (right) IH. Note a larger amount of IH in SVs (bottom) than in IMAs (top). EM-BSEM, ×42–×60 magnification (scale bar: 500 µm and 1 mm, accelerating voltage: 15 kV). Below: Quantitative analysis of 30 pairwise collected IMAs and SVs. Bar graph, *p*-values provided above the bars, Pearson’s chi-squared test with Yates’s correction for continuity; (**B**) Above: VV pattern in bypass grafts (IMAs, top and SVs, bottom) without (left) or with (right) IH. Note the higher VV number in both IMAs and SVs with IH (right). Red arrows indicate VV. EM-BSEM, ×500 magnification (scale bar: 100 µm, accelerating voltage: 15 kV). Below: correlation of VV count and VV density (A/P area per 1 VV, µm^2^) with IH expressed as neointima-to-intima ratio, Spearman’s rank correlation coefficient; and (**C**) Quantitative analysis of the samples from (**A**,**B**). Each dot represents a cross-section of the epoxy resin-embedded blood vessel (one IMA and one SV per patient). Whiskers indicate range, box bounds indicate 25th–75th percentiles, center lines indicate median. *p*-values provided above boxes, Wilcoxon matched-pairs signed rank test. CABG—coronary artery bypass grafting, VV—vasa vasorum, IMA—internal mammary artery, SV—saphenous vein, IH—intimal hyperplasia, and EM-BSEM—embedding and backscattered scanning electron microscopy.

IH was more frequently detected in SVs than in IMAs—21/30 (70.00%) and 11/30 (36.67%) if counting neointima-to-intima ratio ≥5; 22/30 (73.33%) and 12/30 (40.00%) if counting ≥5% stenosis; and 18/30 (60.00%) and 9/30 (30.00%) if counting ≥10% stenosis (Figure 6A). Similar to the animal model, neointima-to-intima ratio strongly correlated with percent stenosis (Spearman’s r = 0.96 and 0.87, respectively, Figure S5), justifying both of these measures to quantify IH. In concert with the balloon angioplasty setting, number and density of VV in CABG surgery conduits significantly correlated with the amount of IH (Spearman’s r = 0.44 and −0.32, respectively, Figure 6B). Relative quantification of IH found that its larger amount in SVs coincided with the higher number and density of VV in these blood vessels as compared with IMAs (Figure 6C). Intriguingly, density of VV per 1% stenosis was higher in SVs than in IMAs, suggesting a substantial importance of adventitial and perivascular vascularisation for the development of IH in SV grafts (Figure S6).

## 3. Discussion

The interactions between vascularisation and inflammation have long been recognised. VV nourish the blood vessel and provide a hierarchical and efficient route for the migration of immune cells into the tunica adventitia and perivascular adipose tissue [1,2,3,4]. In turn, macrophages within the adventitial/perivascular MPO^+^ clusters release a number of pro-angiogenic molecules, such as vascular endothelial growth factor (VEGF), basic fibroblast growth factor (bFGF), and hypoxia-inducible factor (HIF)-1α, which act as paracrine signals to support the existing vessels as well as to initiate and promote neovascularisation [47,48,49]. At physiological conditions, such interconnection between VV and MPO^+^ clusters maintains vascular homeostasis, while cardiovascular risk factors instigate expansion of VV and MPO^+^ clusters, often leading to a desynchronisation and deregulation of angiogenic and immune response.

Here, we employed two animal models and a clinical scenario to investigate which quantitative features (number, area, or density) of VV and MPO^+^ clusters are associated with IH and whether endothelial dysfunction affects the expansion of VV and MPO^+^ clusters. With respect to these tasks, the first animal model was restricted to balloon-induced vascular injury, while the second one was complemented with the repeated injections of CPPs, a trigger of pathological endothelial activation and endothelial-to-mesenchymal transition [42,43,44]. Among available clinical scenarios, we selected conduits for CABG surgery, as: (1) such patients commonly have multiple cardiovascular risk factors; (2) in most cases, this intervention implies the use of both IMAs and SVs to design a bypass, enabling pairwise collection of these blood vessels; and (3) the indicated grafts often suffer from age-related degradation [6,45,46]. We found that vascular injury after balloon-induced endothelial denudation is associated with higher number, area, and density of MPO^+^ clusters which also positively correlated with the amount of immune cells in the tunica adventitia. In concert with these findings, both adventitial and perivascular MPO^+^ clusters increased in number and were enlarged at endothelial dysfunction triggered by regular injections of CPPs. Further, endothelial dysfunction was associated with extended area occupied by adventitial VV, though not with other quantifiable VV features. Notably, number and area of VV and MPO^+^ clusters were well correlated, suggestive of a pathophysiological link between vascularisation and inflammation within the tunica adventitia and perivascular adipose tissue. In a clinical setting, both number and density of VV showed a moderate and statistically significant correlation with the amount of IH; moreover, VV were more frequent in SVs which have been prone to IH in comparison with IMAs. Collectively, our results highlight the quantitative measures to evaluate the correlation between VV networks, MPO^+^ clusters, and IH and testify to the negative impact of excessive adventitial and perivascular vascularisation, proposing an auxiliary mechanism contributing to the higher long-term patency of IMAs in comparison with SVs upon the CABG surgery.

Previous studies have convincingly shown an association of mechanical injury with both IH and adventitial angiogenesis, suggesting an angiogenesis-independent phase of neointimal thickening upon the mechanical injury and next, an angiogenesis-dependent phase that can be potentially prevented by the administration of angiogenesis inhibitors [50]. Upon the balloon injury, the amount of IH and volume of VV networks in rat arteries significantly increased over time, showing linear trajectories from the baseline to the 16th week post-operation [51,52]. Likewise, sequential wire and balloon injury promoted neovascularisation in the adventitia as compared with balloon injury alone [53]. Yet, these studies evaluated only adventitial VV and did not count VV located in the perivascular adipose tissue. Further, some groups evaluated VV density [50,51,53], while others assessed absolute number of VV per section [52]. To the best of our knowledge, the combination of balloon injury and intravenous administration of endothelial dysfunction triggers has not been applied for the assessment of (neo)vascularisation and inflammation in the rat arteries, possibly because rats are more rarely used for the modeling of genetically engineered or induced dyslipidaemia than are mice, rabbits, and swine.

Regarding the CABG surgery conduits, a histological investigation of failed aortocoronary SV grafts revealed ≈10% pre-implantation stenosis, indicating that IH is not necessarily related to vein graft disease and may develop even in the orthotopic location of the conduit [54]. Another histomorphological study found IH in ≈33% of SV and >40% of IMA grafts selected for CABG at the stage of the intervention [55]. Expectedly, pre-implantation stenosis strongly correlated with the number of cardiovascular risk factors [55]. Importantly, VV amount consistently increased along with the extent of the occlusion in the post-implantation period but was not associated with inherent clinical features such as age and gender or with specific locations of the surgical bypass between the coronary artery branches and SV [54]. The large three-dimensional network of VV in the SV reflects relatively high metabolic activity [56,57] and likely contributes to the development of IH upon the pathological conditions [58]. In comparison with SV and coronary artery, IMA contains considerably less VV in its adventitia and perivascular adipose tissue [59,60,61,62,63] that may at least partially explain its pronounced resistance to atherosclerosis and better long-term freedom from IH upon CABG. Yet, other authors proposed that higher patency of no-touch SV grafts, as compared with skeletonised SV [64,65] conduits, is achieved through the retained VV architecture [66] providing higher levels of endothelial nitric oxide synthase, a potent vasodilator [60,67]. 

Both SV [7,8,10] and IMA [60] VV undergo hypertrophic remodeling and gradually lose a hierarchical orientation at the exposure to cardiovascular risk factors, e.g., high-cholesterol diet [7,8,10,61]. Importantly, most of the patients demanding CABG surgery suffer from dyslipidaemia, arterial hypertension, and overweight/obesity, often aggravated by diabetes mellitus or chronic kidney disease [55,68]. Prominent expression of cell adhesion molecules such as VCAM-1 and ICAM-1 in the VV of SV [69] might pave the route for immune cell invasion into the neointima. Taken together, hypoxia, dysregulated innervation, disrupted arterial blood supply, and alterations of the biomechanical conditions because of anatomic relocation of SV can lead to endothelial dysfunction of its VV that may contribute to the migration of leukocytes and development of IH. In accordance with the stronger association of VV number with stenosis of SV in comparison with IMA, the mentioned maladaptation hypothesis might be less relevant for IMA because of its better tensile properties and less developed VV network; however, this assertion needs to be confirmed by subsequent studies. Notably, the proposed scenario seems to be consistent with that which was observed in balloon-injured rat aortas upon the addition of CPPs which trigger the pathological activation of VV endothelium [42,43,44]. In our study, increased VV density was associated with IH in both experimental and clinical models, suggesting an adverse role of excessive adventitial and perivascular vascularisation in the development of IH upon the balloon injury (rats) and at exposure to the cardiovascular risk factors (patients requiring CABG surgery).

The advantages of our study include: (1) two experimental models involving balloon injury alone and combined with intravenous administration of endothelial dysfunction trigger (CPPs); (2) separate analysis of adventitial and perivascular VV and MPO^+^ clusters; and (3) consistency between the results of both experimental models and clinical scenario. The novelty of our study is that: (1) we, for the first time, established a correlation between the amounts of VV and MPO^+^ clusters, also showing the latter as a valuable factor correlating with the amount of intimal hyperplasia; (2) we found that number and area of MPO^+^ clusters well correlate with the density of immune cells in the adventitia, further highlighting MPO^+^ clusters as an informative marker of vascular inflammation; (3) we first showed the pro-angiogenic and pro-inflammatory role of endothelial dysfunction triggers (calciprotein particles) in the context of the expansion of adventitial and perivascular VV and MPO^+^ clusters; and (4) we demonstrated a clear correlation between the density of VV and the extent of pre-implantation intimal hyperplasia in arterial and particularly venous conduits used for CABG surgery. In conclusion, we propose VV density and immune infiltration of the tunica adventitia as the most convenient and appropriate measure to evaluate the pathophysiological role of (neo)vascularisation upon vascular injury, whilst MPO^+^ clusters density may be used for this task specifically in rats. Amount of (neo)vascularisation directly correlates with the extent of vascular inflammation and IH upon vascular injury in rats and with pre-implantation stenosis in CABG patients, a clinical cohort characterised by multiple cardiovascular risk factors. The possible mechanism for these effects is endothelial dysfunction of VV.

## 4. Materials and Methods

### 4.1. Animal Models

#### 4.1.1. Animals

Male Wistar rats weighing 250–300 g and 12–14 weeks of age, provided by the Research Institute for Complex Issues of Cardiovascular Diseases Core Facility, were used for all animal experiments (*n* = 50). Animals were allocated to the polypropylene cages (5 rats per cage) lined with wood chips and had access to the water and food (rat chow) ad libitum. Throughout the duration of the experiment, the standard conditions of the temperature (24 ± 1 °C), relative humidity (55% ± 10%), and a 12 h light/dark cycle were carefully maintained, and the health status of all rats was monitored daily. No randomisation was performed to allocate animals to experimental groups or cages. There were no specific inclusion or exclusion criteria. Experiments were conducted in a blinded fashion. All procedures were carried out conforming to the European Convention for the Protection of Vertebrate Animals used for Experimental and Other Scientific Purposes (Strasbourg, 1986) and were approved by the Local Ethical Committee of the Research Institute for Complex Issues of Cardiovascular Diseases (ethical approval code 57/2019, approved on 15 May 2019).

#### 4.1.2. Animal Surgery

To assess an association between VV expansion, MPO^+^ clusters growth, and IH, we applied an experimental model of rat aorta angioplasty with a coronary angioplasty balloon catheter to inflict a vascular injury. After the induction of anesthesia with a 3% isoflurane, all animals received inhalation anesthesia with a 1.5% isoflurane during the entire time of surgery. Additional anaesthesia included ketamine 7 mg/kg and xylasine 0.6 mg/kg intraperitoneally if sedation was not complete. Topical warm lidocaine hydrochloride (0.02 g/mL) was applied for ameliorating vascular spasm and to ease the insertion of the catheter through the aortic incision. The adequacy of sedation was verified by toe pinch. Briefly, abdominal aorta was punctured in the proximal direction with a 21-gauge needle, DIOR 2.0 × 15 mm balloon catheter with a 0.014 inch guidewire was then inserted into the aortic lumen, and an angioplasty was finally carried out with inflation pressure of 5 atm for 30 s. None of the rats died throughout the entire time of the experiment before the euthanasia. Postoperative painkilling was carried out by subcutaneous administration of buprenorphine (0.1 mg/kg immediately following surgery and twice daily for 3 days post-operation).

#### 4.1.3. Postoperative Interventions

With the aim to investigate whether the endothelial dysfunction enhances VV and MPO^+^ clusters expansion in the context of pre-existing vascular injury, in 10 out of 50 rats balloon angioplasty was followed by daily tail vein injections of either calciprotein particles (CPPs, 900 µL of particles per injection, equal to 600 µg calcium, *n* = 5) or equal volume of physiological saline for 5 days (*n* = 5). CPPs were produced artificially as in our previous work [42,43,44,70]. Five weeks post-operation, all rats were euthanised by an intraperitoneal injection of a sodium pentobarbital (100 mg/kg body weight). Abdominal aortas (≈1 cm length) were immediately excised at the site of injury and dissected into two equal (≈5 mm length) segments. The first segment was fixed in two changes of 10% neutral phosphate buffered formalin (HT501128, Sigma-Aldrich, St. Louis, MO, USA) for 24 h at 4 °C for the further histological and backscattered scanning electron microscopy (EM-BSEM) analysis, while the second segment was snap-frozen in optimal cutting temperature compound (Tissue-Tek, 4583, Sakura, Tokyo, Japan) at liquid nitrogen temperature for the subsequent immunohistochemical or immunofluorescence staining.

#### 4.1.4. Histological and Immunohistochemical/Immunofluorescence Staining

For 40 rats which did not receive tail vein injections upon the angioplasty, formalin-fixed aortic segments were further dehydrated in ascending ethanol series (70, 80, and 95%, 1 h per each, 15058, Electron Microscopy Sciences, Hatfield, PA, USA) and isopropanol (1 h, 190764, Sigma-Aldrich, St. Louis, MO, USA), impregnated and embedded into paraffin (3 changes, 1 h per each, Paraplast REGULAR, 39601006, Leica Biosystems, Wetzlar, Germany), cooled at 4 °C overnight, frozen at −20 °C, and cut (5 µm sections) on a microtome (Microm HM 325, Thermo Scientific, Waltham, MA, USA). To ensure the proper histological examination, we prepared 12 sections, evenly distributed across the entire aortic segment, per slide. Sections were then stained with haematoxylin and eosin (ab245880, Abcam, Cambridge, UK) according to the manufacturer’s protocol and visualised by light microscopy (AxioImager.A1 microscope and EC Plan-Neofluar 20×/0.50 or EC Plan-Neofluar 40×/0.75 M27 objectives, Carl Zeiss, Oberkochen, Germany).

Snap-frozen aortic segments were sectioned (7 µm) on a cryostat (Microm HM 525 and CryoStar NX50, Thermo Fisher Scientific, Waltham, MA, USA) as described above and fixed in acetone for 10 min immediately before the further immunophenotyping of MPO^+^ clusters, which was performed by immunohistochemical staining (Novolink Max Polymer Detection System, RE7280-CE, Leica Biosystems, Wetzlar, Germany) according to the manufacturers’ protocol using the antibodies to a pan-leukocyte marker CD45 (1:200 dilution, ab10558, Abcam, Cambridge, UK), pan-myeloid markers CD11b (1:250, ab133357, Abcam, Cambridge, UK) and MPO (1:100, ab208670, Abcam, Cambridge, UK), pan-macrophage markers F4/80 (1:250, ab100790, Abcam, Cambridge, UK) and CD68 (1:500, ab125212, Abcam, Cambridge, UK), pan-T cell marker CD3 (1:200, ab16669, Abcam, Cambridge, UK), pan-B cell marker CD19 (1:200, MA-32544, Thermo Fisher Scientific, Waltham, MA, USA), and neural tissue markers S100B (1:100, NBP1-87102, Novus Biologicals, Littleton, CO, USA) and NeuN (1:200, NBP1-77686, Novus Biologicals, Littleton, CO, USA). Haematoxylin from the mentioned immunostaining kit was used as a counterstain. Visualisation was conducted as described above. Alternatively, sections were incubated with the antibodies to MPO (1:100, ab208670, Abcam, Cambridge, UK) and CD3 (1:200, ab16669, Abcam, Cambridge, UK) overnight at 4 °C, stained with Alexa Fluor 555-conjugated antibodies (1:250, ab150062, Abcam, Cambridge, UK), and counterstained with 4′,6-diamidino-2-phenylindole (DAPI, 10 µg/mL, D9542, Sigma-Aldrich, St. Louis, MO, USA). Coverslips were mounted with ProLong Gold Antifade (P36934, Thermo Fisher Scientific, Waltham, MA, USA). Slides were examined by confocal laser scanning microscopy (LSM 700, Carl Zeiss, Oberkochen, Germany).

#### 4.1.5. Backscattered Scanning Electron Microscopy

For 10 rats which received tail vein injections of either CPPs or physiological saline after the angioplasty, aortas were postfixed in 1% osmium tetroxide (OsO_4_, 19110, Electron Microscopy Sciences, Hatfield, PA, USA) for 24 h, stained in 2% osmium tetroxide for 40 h, dehydrated in ascending ethanol series (50%, 60%, 70%, 80%, and 95%, two changes per each concentration, 15 min per change), stained in 2% alcoholic uranyl acetate (22400, Electron Microscopy Sciences, Hatfield, PA, USA) for 16 h, dehydrated and degreased in isopropanol (2 h) and acetone (2 h, 179124, Sigma-Aldrich, St. Louis, MO, USA), impregnated with acetone: epoxy resin (EMbed-812, 14120, Electron Microscopy Sciences, Hatfield, PA, USA) mixture (1:1) for 16 h and with epoxy resin for 24 h, and were finally embedded into the fresh epoxy resin at 60 °C for 24 h. Samples were then grinded, polished (TegraPol-11, Struers, Copenhagen, Denmark), and counterstained with Reynolds’s lead citrate (17810, Electron Microscopy Sciences, Hatfield, PA, USA) for 7 min. After a brief washing in double-distilled water, samples were sputter-coated (10 nm thickness) with carbon (EM ACE200, Leica Microsystems, Wetzlar, Germany) and visualised by means of EM-BSEM [71,72] at 15 kV voltage (S-3400N, Hitachi, Tokyo, Japan).

#### 4.1.6. Post-Acquisition Image Analysis

Neointima was defined as an excessive tissue between the endothelial monolayer and internal elastic lamina. Any blood vessels within the adventitia and perivascular adipose tissue (excluding concomitant veins) were considered as VV. Total number and area of VV and MPO^+^ clusters in the examined vessels, further normalised by the area of adventitia and perivascular adipose tissue to calculate VV and MPO^+^ clusters density, as well as the amount of IH were evaluated using the ImageJ software (National Institutes of Health, Bethesda, MN, USA). IH was assessed as neointima-to-intima ratio ≥5 or ≥5% and ≥10% stenosis. Neointima-to-intima ratio was defined as the maximum thickness of neointima (defined as above) divided by the thickness of the intact intima (a normal distance from the apical surface of endothelial cells to the internal elastic lamina). Percent stenosis was quantified as the neointimal area divided by the total area of the vessel lumen. Quantitative analysis was performed in a blinded fashion by two independent pathologists specialising in vascular biology (L.B. and A.K.) who carried out a joint analysis in the case of ≥10% variance between their assessments until reaching a consensus decision.

### 4.2. Clinical Scenario

#### 4.2.1. Patients

The collection of clinical specimens was approved by the Local Ethical Committee of the Research Institute for Complex Issues of Cardiovascular Diseases (ethical approval codes 122/2019 and 123/2019, approved on 30 September 2019), and a written informed consent was provided by all study participants after receiving a full explanation of the study. The investigation was carried out in accordance with the Good Clinical Practice and a latest revision of Declaration of Helsinki (2013). Criteria of inclusion were: (1) CABG surgery because of chronic coronary syndrome and (2) a signed written informed consent to be enrolled. There were no specific criteria of exclusion. In total, we consecutively enrolled 30 patients who underwent CABG surgery upon the admission to the cardiac surgery unit of the Research Institute for Complex Issues of Cardiovascular Diseases (Kemerovo, Russian Federation). IMAs and SVs were pairwise collected from all 30 patients who underwent CABG surgery. In addition, we collected coronary artery adipose tissue to investigate whether MPO^+^ clusters are specific for rats or also exist in humans.

Chronic coronary syndrome [45,73] and comorbid conditions (arterial hypertension [74], chronic heart failure [46], chronic obstructive pulmonary disease [75], asthma [76], chronic kidney disease [77], diabetes mellitus [78], and overweight and obesity [79]) were diagnosed and treated according to the respective guidelines of European Society of Cardiology [45,46,73,74], Global Initiative for Chronic Obstructive Lung Disease [75], Global Initiative for Asthma [76], Kidney Disease: Improving Global Outcomes [77], American Diabetes Association [78], and European Association for the Study of Obesity [79]. Glomerular filtration rate was calculated according to the Chronic Kidney Disease Epidemiology Collaboration (CKD-EPI) equation. Left ventricular ejection fraction was evaluated by means of echocardiography (Sonos 2500 Diagnostic Ultrasound System, Hewlett Packard, Palo Alto, CA, USA). Data on age, gender, smoking status, and pharmacological anamnesis were collected at the time of admission. The detailed characteristics of the study sample are presented in Table 1.

#### 4.2.2. Backscattered Scanning Electron Microscopy Analysis

Immediately upon the excision, IMAs and SVs were fixed in two changes of 10% neutral phosphate buffered formalin for 24 h at 4 °C and prepared for EM-BSEM analysis [71,72] as described above.

#### 4.2.3. Immunofluorescence Examination

Immediately upon the excision, coronary artery adipose tissue was snap-frozen, sectioned, and fixed in acetone for 10 min as described above. Upon the permeabilisation with Triton X-100 (97063-864, VWR, Radnor, PA, USA) for 10 min, sections were incubated with the antibodies to MPO (1:100, ab208670, Abcam, Cambridge, UK) or CD68 (1:200, ab955, Abcam, Cambridge, UK) overnight at 4 °C, stained with Alexa Fluor 555-conjugated antibodies (1:250, ab150062, Abcam, Cambridge, UK), and counterstained with DAPI (10 µg/mL, D9542, Sigma-Aldrich, St. Louis, MO, USA). Coverslips were mounted with ProLong Gold Antifade (P36934, Thermo Fisher Scientific, Waltham, MA, USA). Slides were examined by confocal laser scanning microscopy (LSM 700, Carl Zeiss, Oberkochen, Germany).

#### 4.2.4. Post-Acquisition Image Analysis

Total number and area of VV in IMAs and SVs, further normalised by the area of adventitia and perivascular adipose tissue to calculate VV density, as well as the amount of IH in these blood vessels were evaluated as described above.

### 4.3. Statistical Analysis

Statistical analysis was performed using GraphPad Prism 8 (GraphPad Software, San Diego, CA, USA). For descriptive statistics, data were represented by the proportions or by the median, 25th and 75th percentiles, and range. Proportions were compared using Pearson’s chi-squared test with Yates’s correction for continuity. Independent and paired groups were compared by Mann–Whitney U-test or Wilcoxon matched-pairs signed rank test, respectively. Correlation analysis was conducted using Spearman’s rank correlation coefficient. *p*-values ≤ 0.05 were regarded as statistically significant.

## Figures and Tables

**Table 1:** Clinicopathological features of the patients who underwent CABG surgery.

Clinicopathological Features	N (%)/Median (Interquartile Range)
Age and gender	
Male gender	21/30 (70.00%)
Age, years	63.50 (59.75–68.00)
Comorbid conditions	
Arterial hypertension	30/30 (100.00%)
Chronic heart failure	30/30 (100.00%)
Chronic obstructive pulmonary disease or asthma	1/30 (3.33%)
Smoking	12/30 (40.00%)
Chronic kidney disease	7/30 (23.33%)
Diabetes mellitus	8/30 (26.67%)
Overweight	11/30 (36.67%)
Obesity	15/30 (50.00%)
Quantitative parameters	
Body mass index, kg/m^2^	29.70 (26.70–31.80)
Glomerular filtration rate, mL/min/1.73 m^2^	85.50 (71.50–99.25)
Left ventricular ejection fraction, %	62.00 (52.75–65.60)
Number of affected coronary arteries	3.00 (2.00–3.00)
Pre-treatment	
Antiplatelet drugs	24/30 (80.00%)
Beta blockers	18/30 (60.00%)
Angiotensin-converting enzyme inhibitors	11/30 (66.67%)
Statins	24/30 (80.00%)
Nitrates	0/30 (0.00%)
Angiotensin receptor II blockers	11/30 (36.67%)
Aldosterone antagonists	2/30 (6.67%)
Calcium channel blockers	15/30 (50.00%)
Diuretics	3/30 (10.00%)
Anticoagulants	2/30 (6.67%)

## Data Availability

The data presented in this study are available on request from the corresponding author. The data are not publicly available due to the patients’ concerns.

## Supplementary Materials

The following supporting information can be downloaded at: https://www.mdpi.com/article/10.3390/ijms232012156/s1.
