# Supplementary material for: Excessive Adventitial and Perivascular Vascularisation Correlates with Vascular Inflammation and Intimal Hyperplasia
Source: Int J Mol Sci. 2022 Oct 12;23(20):12156. doi: 10.3390/ijms232012156 (PMC9603343; doi:10.3390/ijms232012156)
Supplement: Supplementary file 1 [file ijms-23-12156-s001.zip › Legends to Supplementary Figures.pdf]

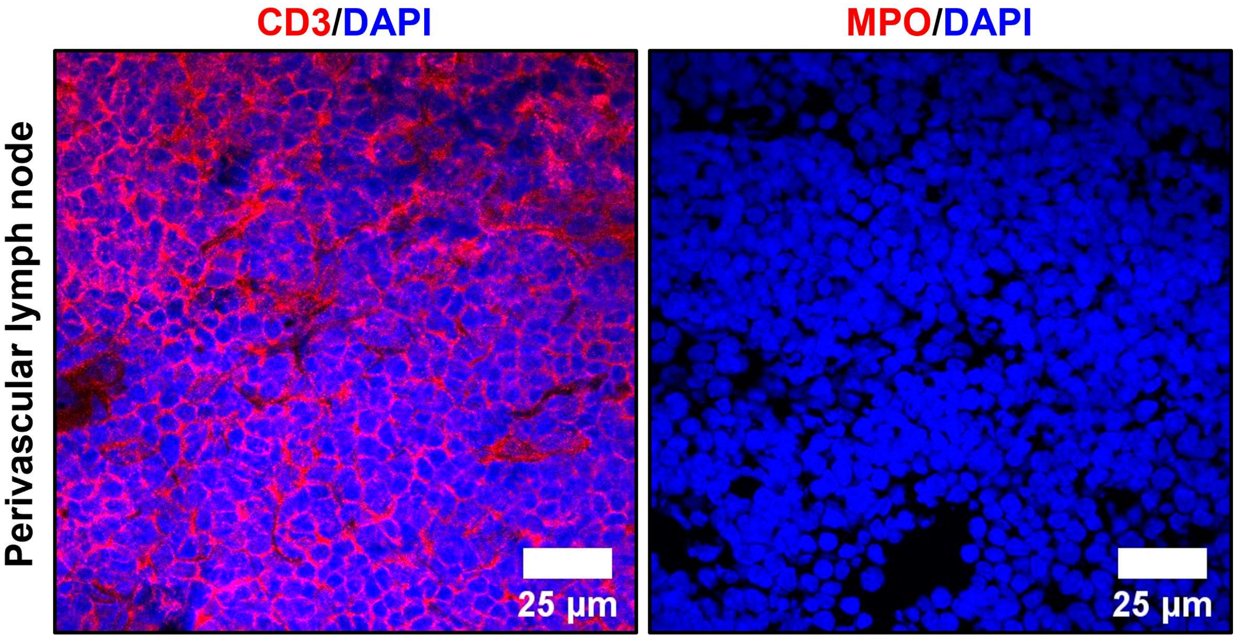

Figure S1. Perivascular lymph node positive for CD3 and negative for MPO (control for antibodies used for the immunohistochemical and immunofluorescence staining in Figure 1A and 1B). DAPI nuclear counterstaining. MPO – myeloperoxidase, DAPI – 4′,6-diamidino-2-phenylindole.

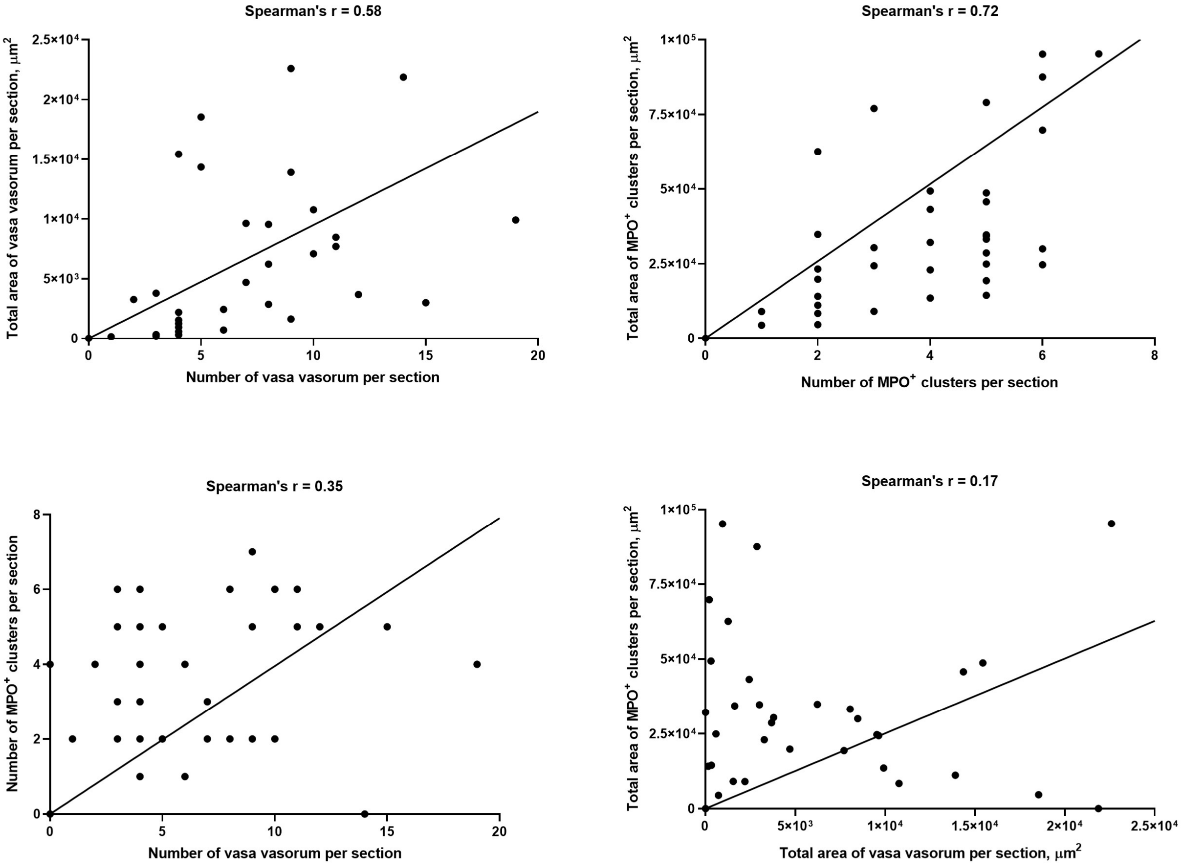

Figure S2. Correlation plots showing a strong correlation between number and area in both VV and MPO^+ clusters (top) as well as moderate correlation between numbers and areas of VV and MPO^+ clusters (bottom). Spearman's rank correlation coefficient. VV – vasa vasorum, MPO – myeloperoxidase.

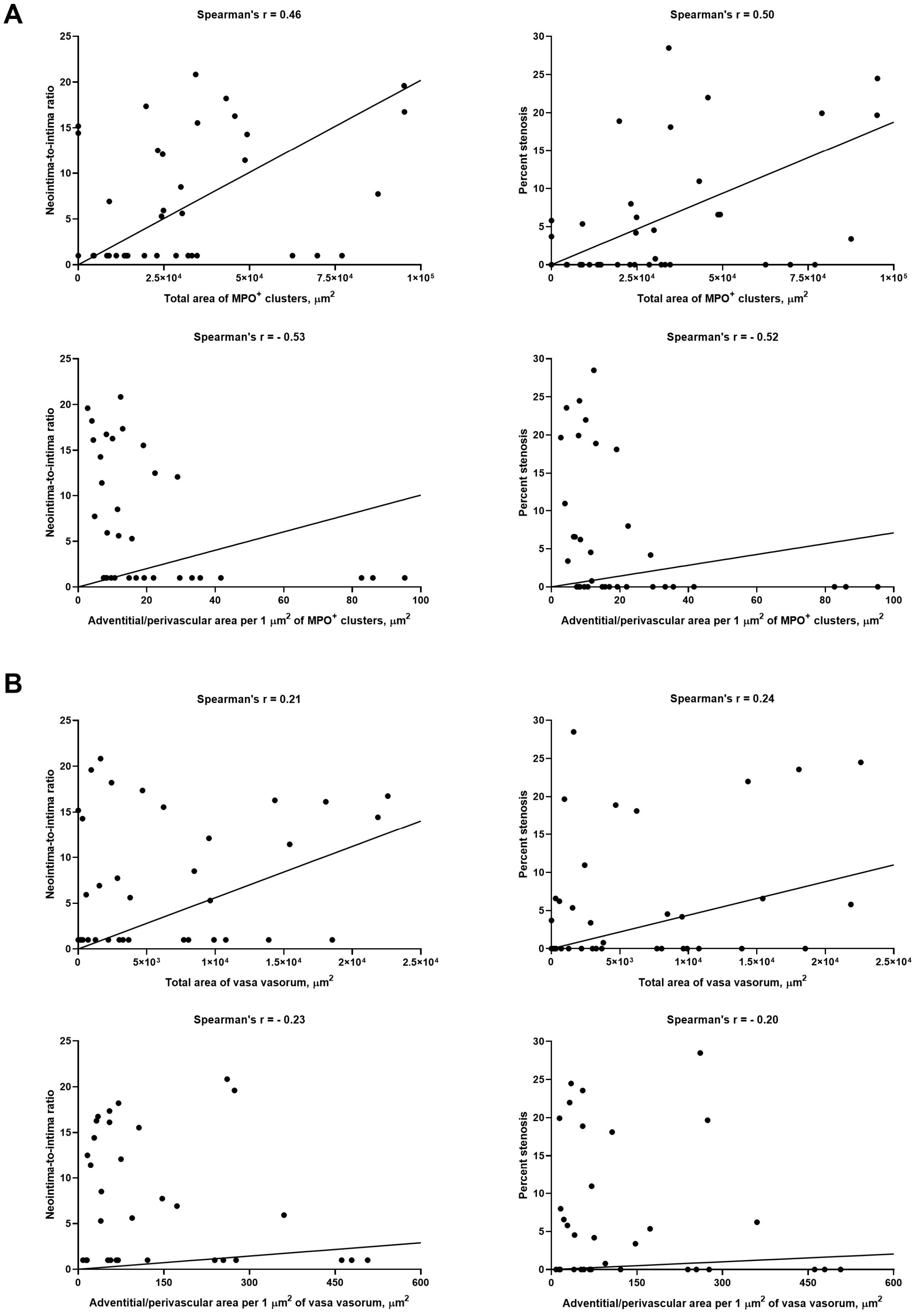

Figure S3. Correlation plots demonstrate a moderate but statistically significant correlation of total area (top) and density (bottom) of both MPO$^+$ clusters (**A**) and VV (**B**) with intimal hyperplasia measured by neointima-to-intima ratio (left) and percent stenosis (right). Spearman's rank correlation coefficient. VV – vasa vasorum, MPO – myeloperoxidase.

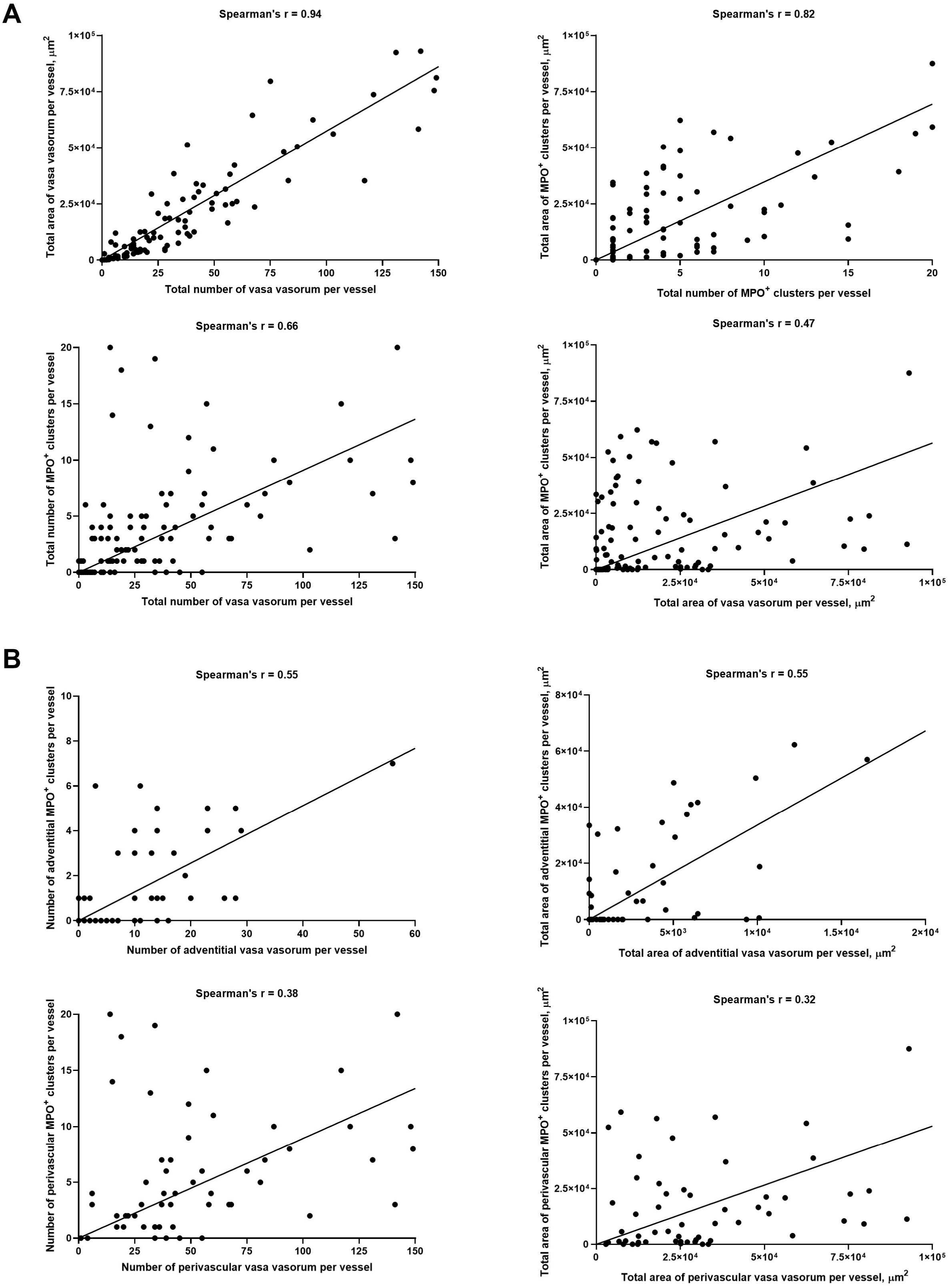

Figure S4. A. Replication model confirms a strong correlation between number and area in both VV and MPO^+ clusters (top) and moderate correlation between numbers and areas of VV and MPO^+ clusters (bottom). **B.** Numbers and areas of VV and MPO^+ clusters well correlate regardless of their anatomical location (tunica adventitia, top or perivascular adipose tissue, bottom). Spearman's rank correlation coefficient. VV – vasa vasorum, MPO – myeloperoxidase.

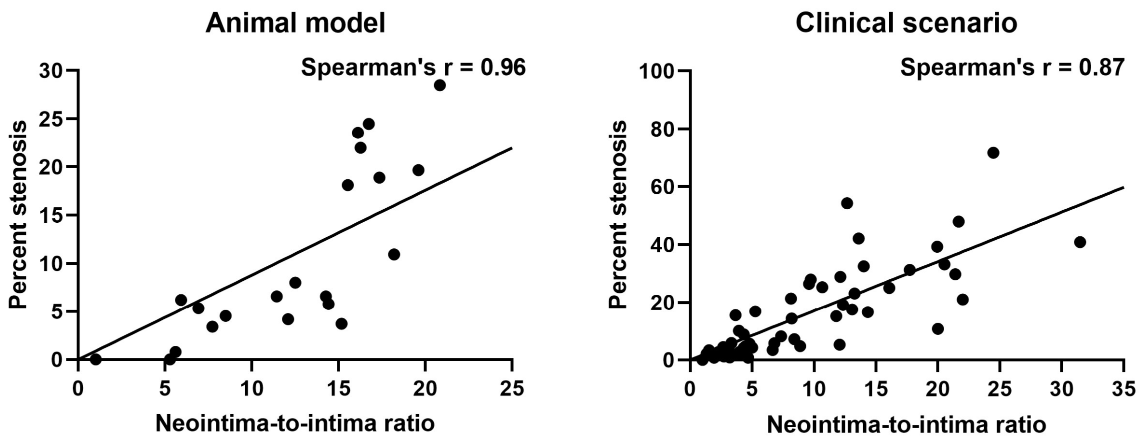

Figure S5. Correlation plots indicate a strong correlation between both measures of intimal hyperplasia (neointima-to-intima ratio and percent stenosis).

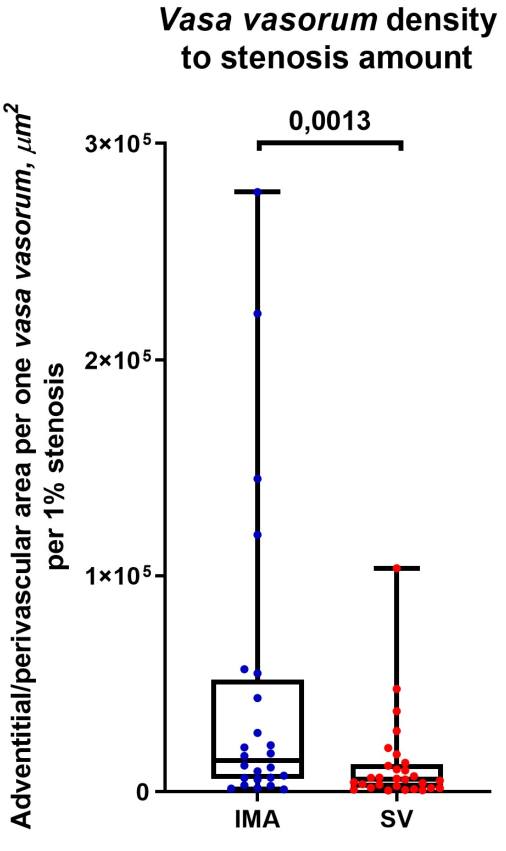

Figure S6. Density of VV (i.e., adventitial and perivascular area per one vasa vasorum) per 1% stenosis is significantly higher in SVs as compared with IMAs. Each dot represents a cross-section of the epoxy resin-embedded blood vessel (one IMA and one SV per patient). Whiskers indicate range, boxes bounds indicate 25th–75th percentiles, center lines indicate median. P value provided above boxes, Wilcoxon matched-pairs signed rank test. VV – vasa vasorum, SVs – saphenous veins, IMAs – internal mammary arteries.
