# Supplementary figures and images for: Excessive Adventitial and Perivascular Vascularisation Correlates with Vascular Inflammation and Intimal Hyperplasia
Source: Int J Mol Sci. 2022 Oct 12;23(20):12156. doi: 10.3390/ijms232012156 (PMC9603343; doi:10.3390/ijms232012156)

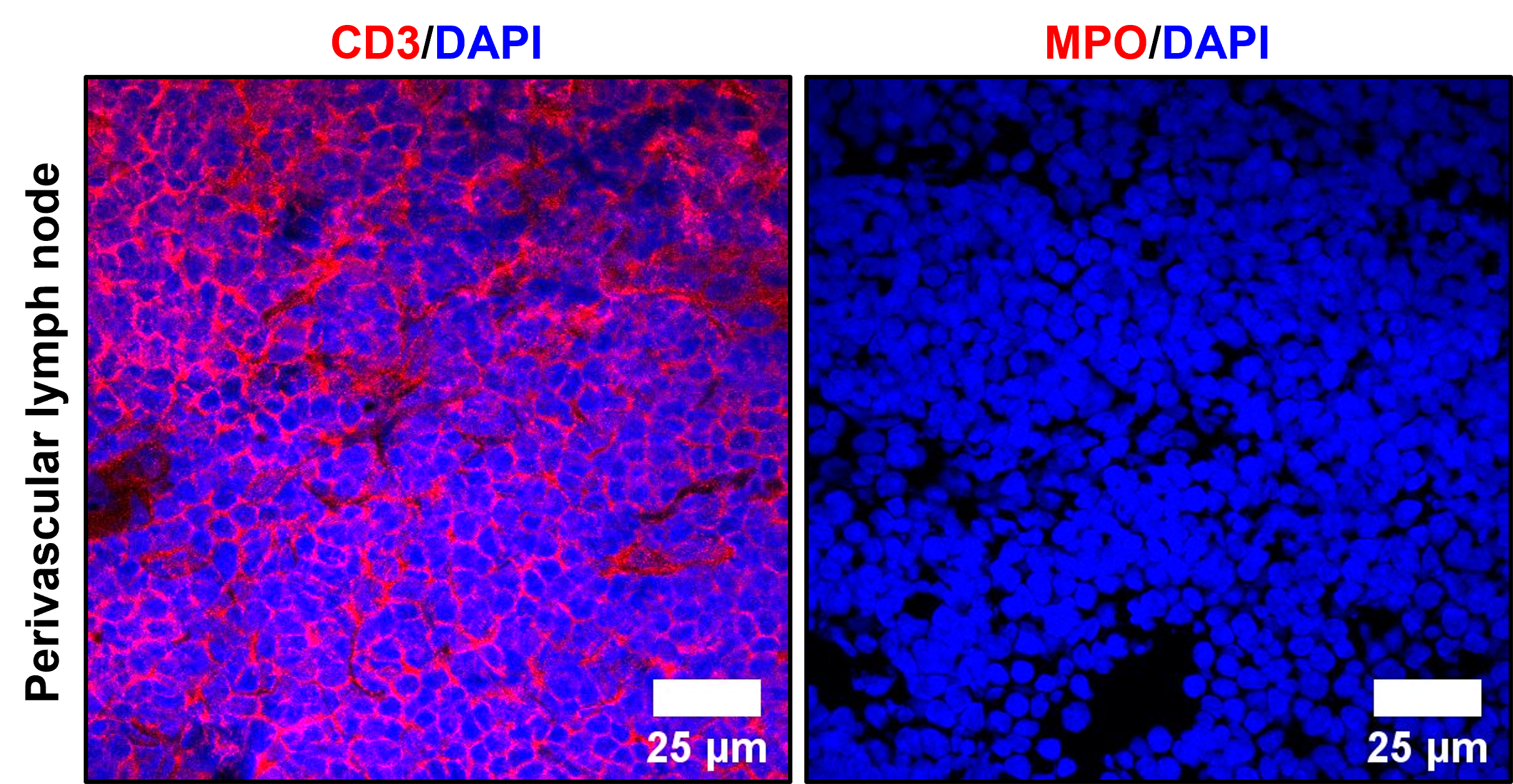

Supplement: Supplementary file 1 [file ijms-23-12156-s001.zip › Figure S1.tif]

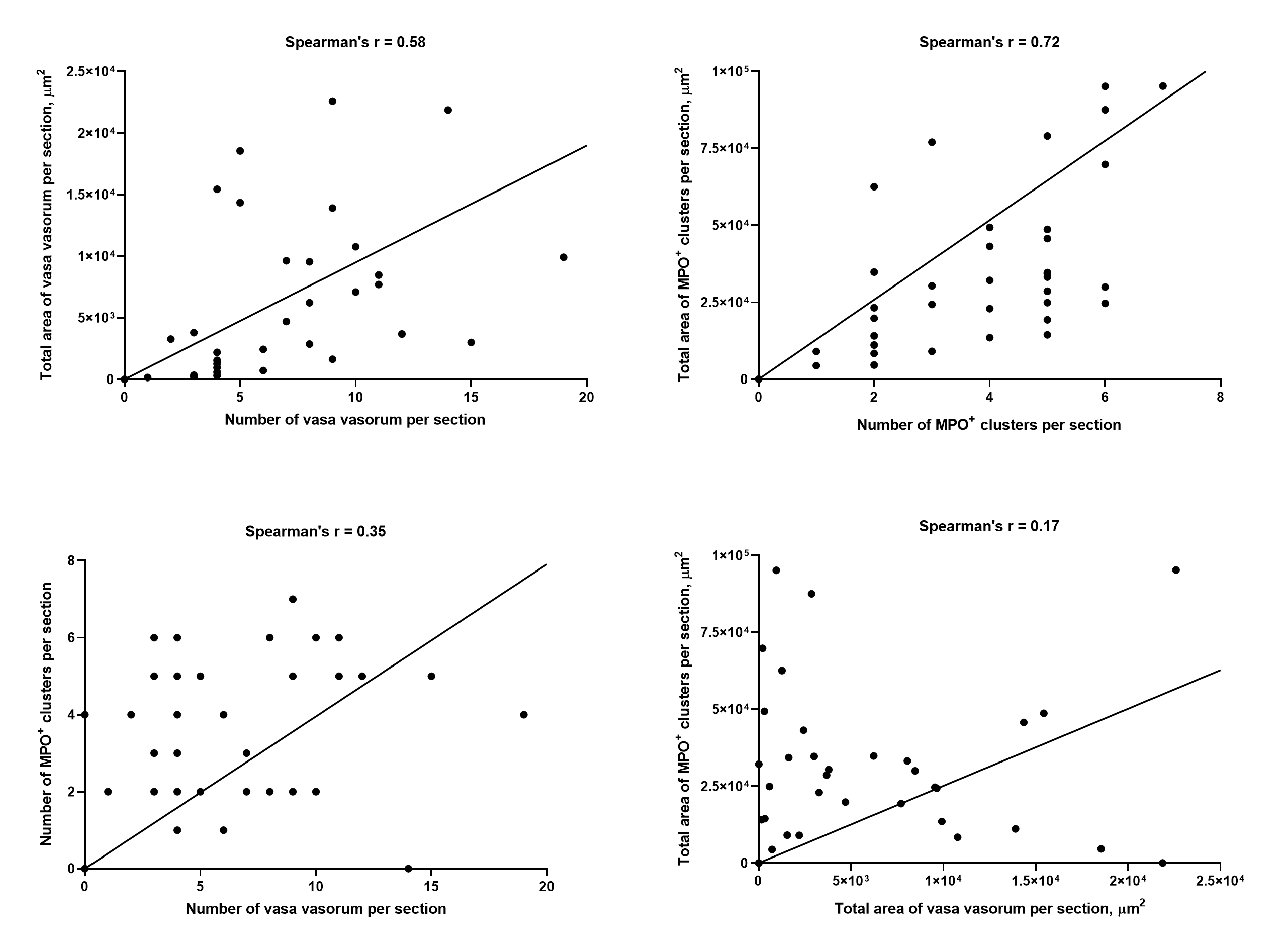

Supplement: Supplementary file 1 [file ijms-23-12156-s001.zip › Figure S2.tif]

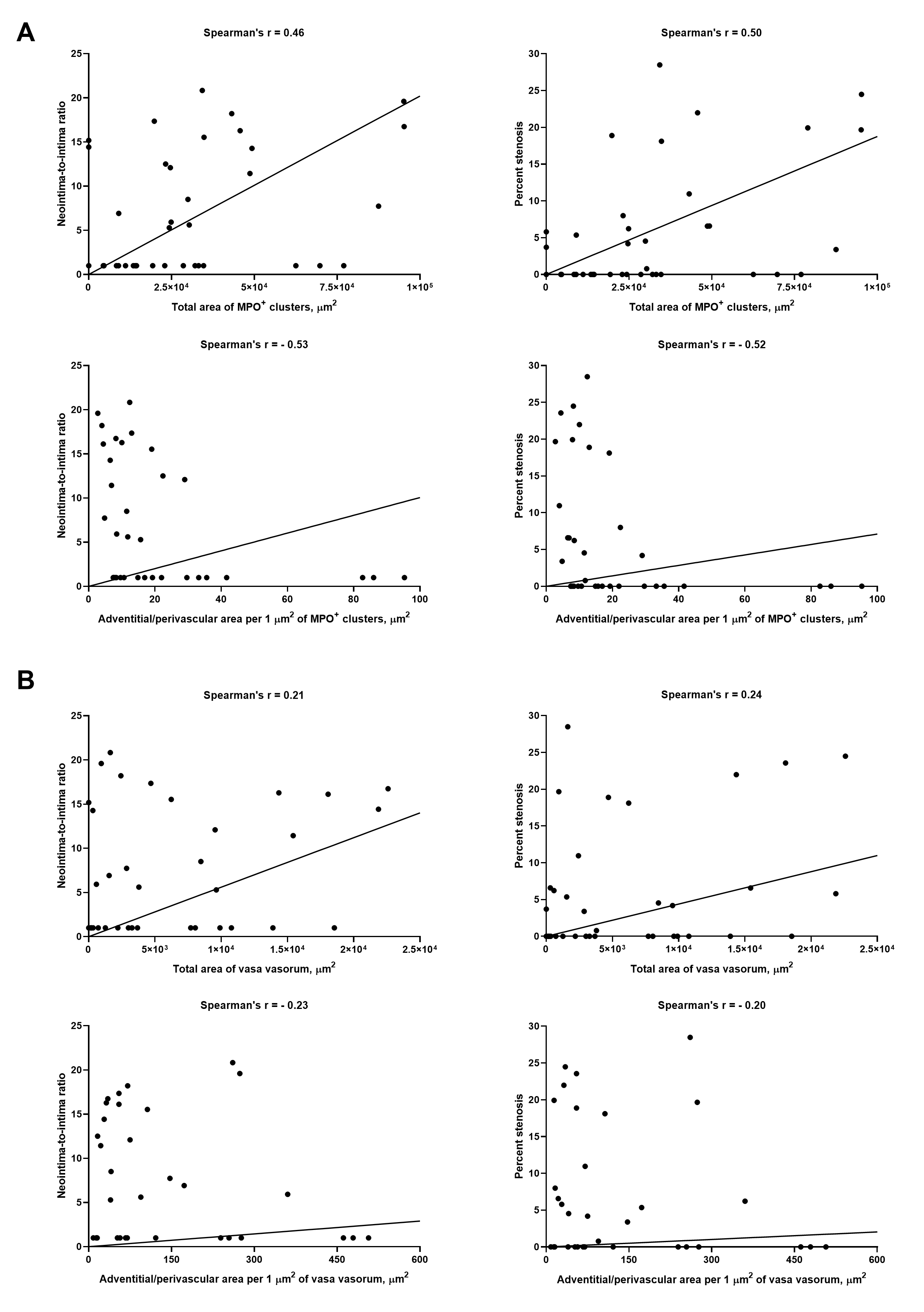

Supplement: Supplementary file 1 [file ijms-23-12156-s001.zip › Figure S3.tif]

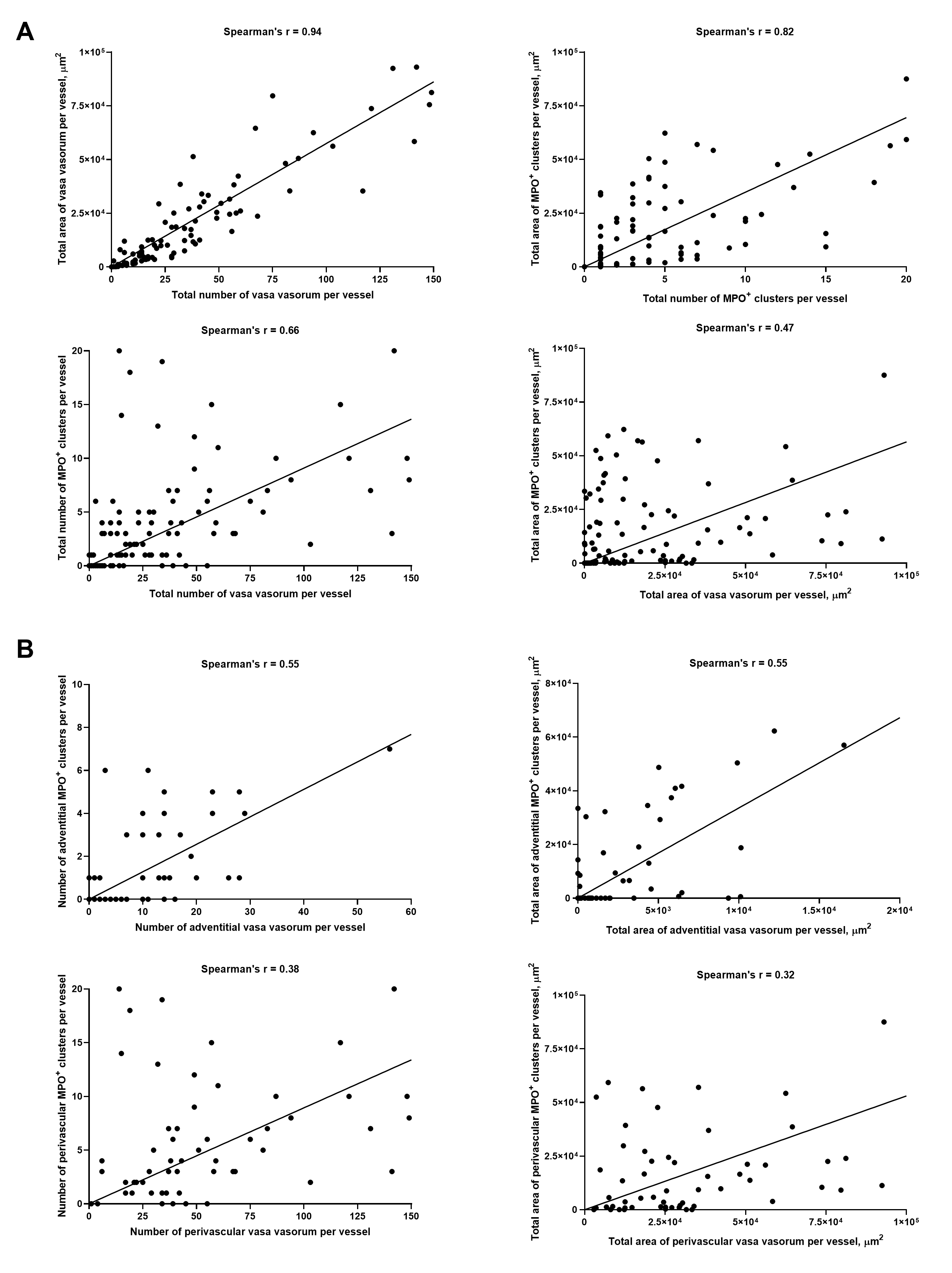

Supplement: Supplementary file 1 [file ijms-23-12156-s001.zip › Figure S4.tif]

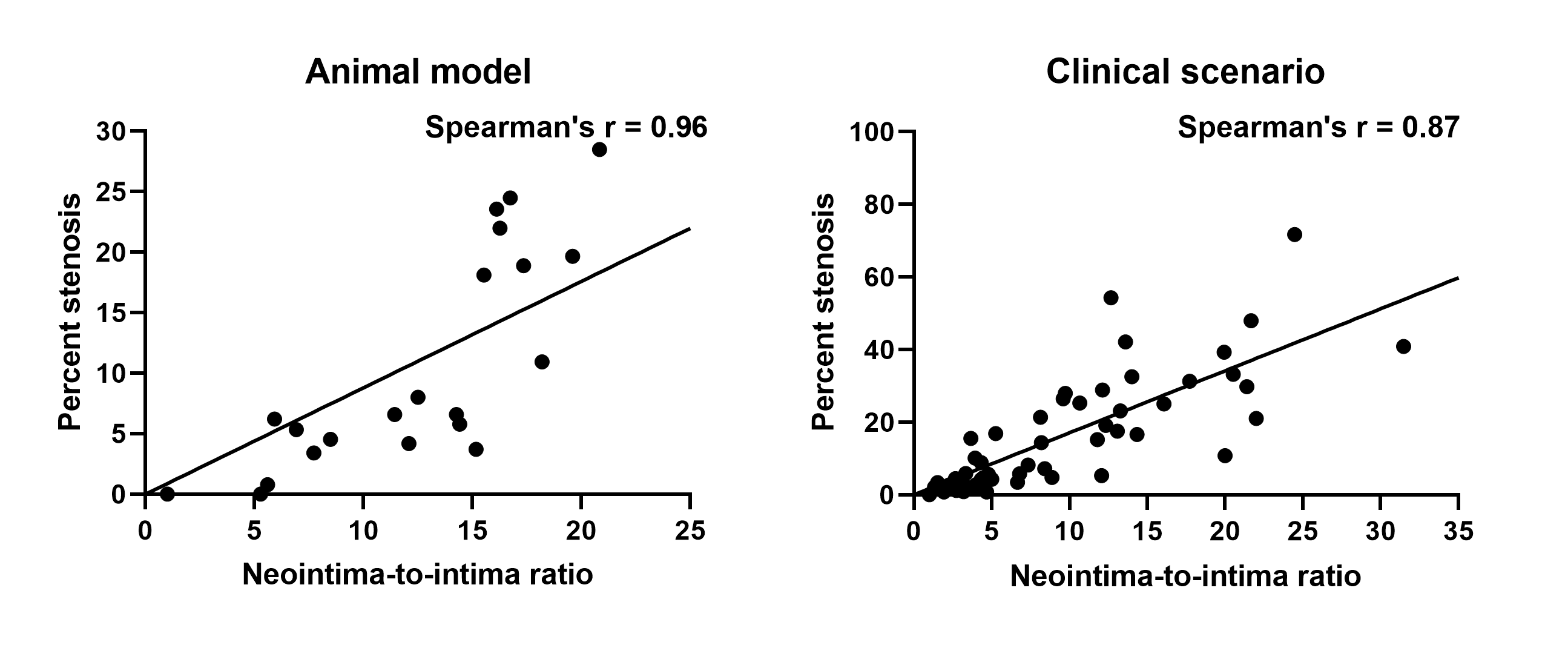

Supplement: Supplementary file 1 [file ijms-23-12156-s001.zip › Figure S5.tif]

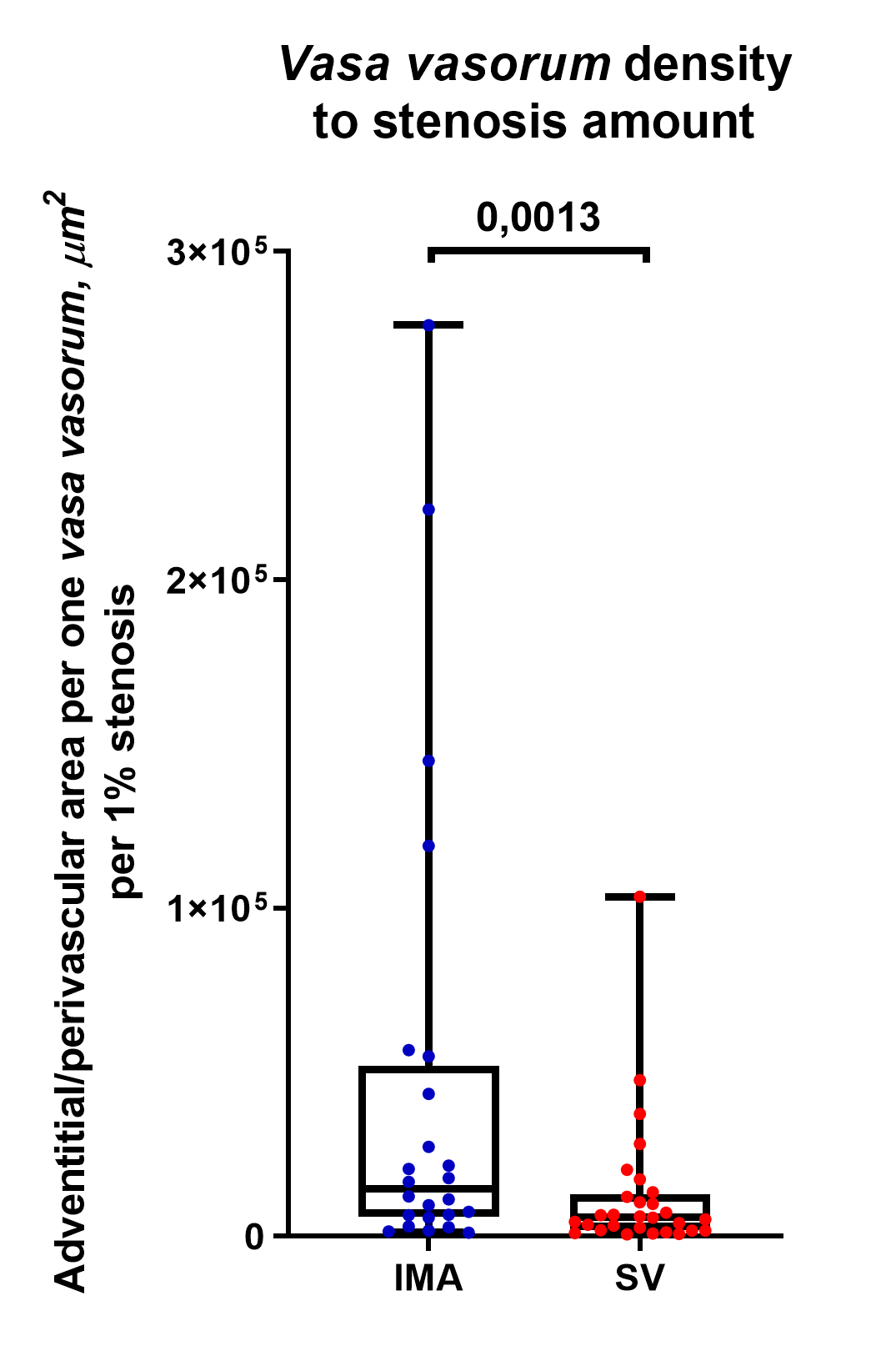

Supplement: Supplementary file 1 [file ijms-23-12156-s001.zip › Figure S6.tif]
